# Evaluation of an Online Patient Education Program for Children and Young People with ME/CFS and their Parents within the BAYNET FOR MECFS Study

**DOI:** 10.1055/a-2773-9655

**Published:** 2026-01-06

**Authors:** Franca Keicher, Julia Thomann, Jana Erlenwein, Mara Schottdorf, Karolina Wiejaczka, Nils Lennart Reiter, Nadine Scholz-Schwärzler, Barbara Vogel, Silvia Stojanov, Silvia Augustin, Milica Saramandic, Robert Jaeschke, Kristina Dettmer, Stephanie Englbrecht, Linda Schanz, Veronika Dodel, Charlotte Zipper, Nicole Schieweck, Gundula Ernst, Uta Behrends, Juliane Spiegler

**Affiliations:** 1 Department of Children and Adolescent Psychiatry, Psychotherapy, and Psychosomatics, University Hospital Würzburg, Würzburg, Germany; 2 Department of Pediatrics, University Hospital Würzburg, Würzburg, Germany; 3 Alice-Salomon University of Applied Sciences Berlin, Berlin, Germany; 4 PhysioBib GbR, Berlin, Germany; 5 MRI Chronic Fatigue Center for Young People, Center for Pediatric and Adolescent Medicine, Technical University of Munich, Munich DE, Germany; 6 Department of Orthopedics and Sports Orthopedics, Physical Therapy, University Hospital Rechts der Isar, Technical University of Munich, Munich DE, Germany; 7 Rehabilitation Centre for Children with Respiratory Diseases, Fachkliniken Wangen, Wangen DE, Germany; 8 Parent Initiative for Children and Adolescents with ME/CFS Munich e.V., Munich DE, Germany; 9 Department of Medical Psychology, Hannover Medical School, Hannover, Germany

**Keywords:** online education program, ME/CFS, pediatrics, ModuS, CYP, parents

## Abstract

**Background:**

Myalgic encephalomyelitis/chronic fatigue syndrome (ME/CFS) poses challenges for affected children and young people (CYP) and their parents. There is often a lack of knowledge about the illness. Education programs can help address this by providing knowledge and supporting the independent management of the condition. For this reason, two online education programs—one for affected CYP and one for their parents—were developed, implemented, and evaluated in terms of acceptance, format, and benefits.

**Methods:**

A total of 24 CYP with ME/CFS, aged of up to 20 years, and their parents were recruited for this study. Of these 22 CYP with ME/CFS and 20 parents participated in the online education program. After development and conduct of the programs, six affected CYP were interviewed using written questions, which were answered via an audio device. Furthermore, six semi-structured interviews were obtained with parents. All parents also received an online questionnaire to evaluate the program. Data were analyzed using both quantitative and qualitative methods.

**Results:**

Both CYP and their parents expressed overall satisfaction with the program, highlighting aspects such as knowledge acquisition or reinforcement and, importantly, the opportunity to connect with other affected CYP or their parents. The online format was also perceived very positively.

**Discussion:**

The online education program met the expectations and needs of both affected CYP and parents regarding content and format. It facilitated exchange and provided practical knowledge. In this format, the online program appears to be a valuable component of care for those affected.

## Background

Myalgic encephalomyelitis/chronic fatigue syndrome (ME/CFS) is a chronic illness with an etiology that is not yet fully understood. 1 Affected people experience a variety of symptoms such as fatigue, sleep difficulties, and post-exertional malaise (PEM), a worsening of symptoms often occurring after minimal physical, cognitive, or emotional exertion. 2 3 Furthermore, orthostatic dysregulation, including postural orthostatic tachycardia syndrome (POTS), cognitive deficits, and pain, can be part of the clinical picture. 1 2 4 This, along with the associated impairment of social participation in family, school, and recreational activities, often leads to a significant reduction in the quality of life for those affected. 5 Due to the lack of a causal treatment, therapy relies on managing individual symptoms. To teach these strategies to affected individuals and empower them to self-manage the illness, patient education is recommended as part of their care. 6 To date, there is a lack of standardized education programs for children and young people (CYP) with ME/CFS. However, positive experiences have been reported with education programs for other medical conditions, such as bronchial asthma or atopic dermatitis. 7 8 9 Studies on education programs have reported positive effects on quality of life, as well as a reduction in relapses and disease-specific symptoms. 7 9 10 11 A commonly used, standardized approach applicable to various chronic illnesses is the modular education program ModuS by the “Kompetenznetzwerk Patientenschulung (KomPaS)” (competence-network patient education). 12 The education concept also entails an adapted form for parents 7 as they play key roles as both caregivers and therapists for their children and can themselves be significantly burdened by chronic illnesses. 1 13 14 Due to the limited mobility of affected CYP with ME/CFS, for whom overexertion can lead to PEM, 1 and the constrained time resources of their families, a traditional in-person education program is not a viable option for this target group. Therefore, within the BAYNET FOR ME/CFS study, two standardized online education programs based on the ModuS concept, one for individuals with ME/CFS aged up to 20 years and one for their parents, were developed, conducted, and evaluated. The goal was to investigate the following questions:

1. What is the level of acceptance of the online education program by the affected individuals and parents?
2. How do both groups evaluate the online education program in terms of format, content, and benefit?

## Methods

### Ethical Considerations

The Ethics Committee of Julius-Maximilians-University approved the conduct of the online education programs evaluation on August 11, 2023 (approval number 71/23-sc).

### Study Population

Recruited study participants included 24 CYP aged up to 20 years and 24 parents of this group of CYP. The CYP cohort was diagnosed with ME/CFS at the Munich Chronic Fatigue Center for Young People (MCFC) at the Technical University of Munich within the BAYNET FOR MECFS joint project framework. After being diagnosed with ME/CFS, CYP and their parents were invited to participate in this project and, upon agreement, were automatically enrolled in the education programs.

### Online Education Program

The development and conceptualization of the online education programs were performed by an interdisciplinary study team including members with ME/CFS from patient organizations, experienced physicians, psychologists, physiotherapists, and occupational therapists, within the BAYNET FOR MECFS joint project framework. Initially, the needs of the affected CYP and their parents were identified through consultations with experts, support groups, and a literature review. The modular education program concept ModuS 12 served as a template for the two programs. After piloting, the education programs were revised and completed. The structure and content of the final education programs are shown in the Supplementary Table S1 and Supplementary Table S2 (for more detailed information, see Keicher et al 15 ). The sizes for CYP and parent groups were limited to 12. Prior to the online education program, participants were sent the working material by regular post. Furthermore, all participants were referred to the programs' website which included information about the etiology and management of ME/CFS as well as links to resources. Both the online education programs for CYP as well as for their parents were conducted twice between May and September 2023.

### Evaluation

To evaluate the education program for CYP with ME/CFS, interviews were conducted with three participants who were mildly to moderately affected (with attendance at school) and three participants who were severely affected (without school attendance). Participants were randomly selected after being divided into subgroups based on illness severity and gender. We aimed for a balanced gender ratio within the two subgroups of illness severity. In cases of cancellation or missing responses from a selected participant, the selection process was repeated with the remaining participants. Over a period of 2 weeks, four sets of questions comprising a total of 19 items were sent (see Supplementary Table S3 , available in the online version only). Participants could respond using an audio device and upload their answers via a link to the clinic's internal data exchange platform, FEX. Furthermore, epidemiological data such as age and gender, duration of illness, severity of illness, and daily activity using the MCFC activity scale 16 17 and the Bell score 18 were collected from all individuals with ME/CFS. This approach was selected to accommodate the patients' limited energy and concentration capacity, thereby aiming to prevent PEM.

The evaluation of the parent education program included a questionnaire and interviews with three parents of mildly to moderately affected CYP (able to attend school/educational facilities) and three parents of severely to extremely affected CYP (unable to attend school). The interviews were conducted via Zoom using a semi-structured interview guide (see supplement) and recorded.

The quantitative questionnaire data were analyzed using the SPSS software (Version 29.0.1.1) through descriptive analysis such as means and standard deviations.

The qualitative analysis of the interviews was conducted using the MAXQDA software 19 following content analysis according to Kuckartz. 20 In this process, categories were formed deductively and inductively by three individuals independently—a pediatrician, a psychologist, and an occupational therapist. These categories were then discussed and agreed upon. After reaching a consensus, the transcripts were reanalyzed using the new category system.

## Results

### Online Education Program for CYP with ME/CFS

Of 24 CYP with ME/CFS recruited 22 took part in the education program (see Table 1 ). One individual did not participate due to having already taken part in the pilot study, while another opted out of all study activities for unspecified reasons.

**Table 1:** Overview of study participants with ME/CFS ( 1 0 = lowest activity level to 6 = highest activity level, 2 level of restriction 0 = bedridden to 100 = unrestricted functioning)

	Recruited in study	Participated in education program	Participated in interviews
**Number of participants**	24	22	6
**Age**	16,0	16,0	16,0
**Gender ratio (female:male)**	19:5	17:5	6:0
**Duration of illness (months)**	18.8	18.5	14.5
**Severity ratio (low/moderate:high)**	15:9	14:8	3:3
** MCFC activity scale ^1^**			
• **Self-sufficiency**	4.7	4.6	4.2
• **Physical activity** • **Mental activity**	4.1 2.9	4.0 2.9	4.0 3.0
• **Social contacts**	3.8	3.7	3.8
• **School and education**	2.3	2.3	2.7
** Bell score ^2^**	34.2	34.1	36.7

Abbreviations: MCFC, Munich Chronic Fatigue Center for Young People; ME/CFS, myalgic encephalomyelitis/chronic fatigue syndrome.

The average age of the six CYP who completed the interview questions was 16.0 years, and all individuals were female. The desired balanced gender distribution could not be achieved due to participant cancellations and incomplete response blocks.

From the responses, the following six main categories were formed: characteristics of participants, education program resources, (online) format, outcome, overall evaluation, and recommendation (see Supplementary Fig. S1 , available in the online version only).

#### Characteristics of Participating CYP

Most CYP expressed the expectation of gaining new information from the program, with the “hope of learning tips that I did not know yet.” Other expectations included learning about disease management and exchanging experiences with other affected individuals to “encounter encounter more understanding” and improving the management of the illness. Many reported having significant prior knowledge, acquired through experiences such as hospital stays, medical consultations, or independent reading.

#### Education Program Resources

The provided materials were generally positively evaluated; however, opinions differed regarding specific materials. Overall, it was evident that due to the diversity of materials, each CYP evaluated some of it helpful at least to some extent. While one person accepted the program package well, saying, “The envelopes that were included in the program were made with a lot of love, and I thought that was very nice,” another person mentioned, “I honestly couldn't really use the program materials we received at home, so it just lay somewhere in my room,” but at the same time said, “what I really liked, (…) was that we received all the PowerPoint presentations afterward, so you could read through them again.” The facilitators of the program were mostly well-rated as supportive and the technology was perceived as posing no issues. Regarding content, the affected CYP expressed satisfaction with the selection and explanation of the topics.

#### (Online) Format

The online format of the program for CYP was highly positively evaluated “because you didn't have to spend energy on traveling,” and “it was simply accessible, you didn't have to be physically present, so you could almost always participate, even when you weren't feeling well.” The option to turn off the screen was mentioned multiple times as a positive feature, although it was also considered a barrier to interaction at times: “yes, I just found it unfortunate that many, since I often had the camera on, many didn't manage to turn on the camera (…).” The structure of the format including various breaks with guided relaxation exercises was well received.

#### Overall Evaluation

Overall, the evaluation of the program for CYP by the participating individuals was very positive. The group size was perceived as comfortable by all respondents except one person, who would have preferred a smaller group. However, the group atmosphere was unanimously perceived as extremely positive: “Even during the program, everyone was very polite, and people wanted to help each other as much as possible.” This led to fruitful exchanges between participants not only during the program, “to get to know the other people. I thought that was really a great and important aspect of the whole thing,” but also through their initiative to connect further in chat groups with other participants “where we can still exchange ideas.”

Improvement of suggestions included communicating via allocated accounts rather than via email, providing tips for interacting with others when being affected by ME/CFS, incorporating more research aspects and offering a greater variety of topics. This included the areas of brain fog, concentration issues, and nutrition. Some persons indicated that they would have preferred less repetition, “some topics shouldn't be repeated so often because sometimes I felt that things were discussed very, very long and very often.”

#### Outcome

Even though many CYP, already possessing good prior knowledge, reported only modest knowledge gain, the majority of respondents indicated that they learned new information about the illness and how to manage the symptoms “because you still got some information about it.” Participants also mentioned a newfound awareness of the importance of “pacing” which was highlighted as a crucial result of the program: “So my everyday life has changed through the program in the sense that I now try to implement ‘pacing’ a bit more. I mean, before I already knew roughly how it works and such, but really, with a weekly planner and thinking more about it (...) that has changed. (...).” Other positive effects were reported regarding learning and applying new relaxation techniques and implementing the experiences shared by other participants. The sense of belonging the CYP experienced in the program was often considered particularly helpful: “(…) because you also just feel less alone because that is often such an aspect because in my environment, I don't really know people with the illness, and then it's just very pleasant that you feel taken seriously and that you get to know other people of the same age.” Additionally, some CYP viewed the education program as helpful in managing their own illness. The program also had an impact on social integration. One participant stated after the program to have “found the courage to get myself a wheelchair so that I can participate in outings where more walking would be required again.”

#### Recommendation

In all interviews, CYP expressed their intention to recommend the program. One person stated: “If a friend of mine were affected, I would definitely recommend the program to them and encourage them. It doesn't make you healthy, but it definitely helps.”

### Online Education Program for Parents of Children and Young People with ME/CFS

Of 24 parents recruited for the study, 20 participated in the education program and 6 were interviewed (see Table 2 ). One parent did not participate due to prior involvement in the pilot program, two declined because their child was over 18 years old, and one did not participate in any study activities without providing an explanation.

**Table 2:** Overview of study participants who were parents of children/adolescents with ME/CFS

	Recruited in study	Participated in education program	Participated in interviews
**Number of participants**	24	20	6
**Children's age**	16.0	15.6	13.7
**Children's gender ratio (female:male)**	19:5	15:5	4:2
**Children's duration of illness (months)**	18,8	16,8	12,3
**Children's severity ratio (low/moderate:high)**	15:9	12:8	3:3
** Children's MCFC activity scale 1**			
• **Self-sufficiency**	4.7	4.6	4.8
• **Physical activity**	4.1	4.1	4.2
• **Mental activity**	2.9	2.8	2.8
• **Social contacts**	3.8	3.6	3.8
• **School and education**	2.3	2.1	2.0
** Bell score ^2^**	34.2	31.5	28.3

Abbreviations: MCFC, Munich Chronic Fatigue Center for Young People; ME/CFS, myalgic encephalomyelitis/chronic fatigue syndrome.

Notes: 1 0 = lowest activity level to 6 = highest activity level.

2 Level of restriction 0 = bedridden to 100 = unrestricted functioning.

#### Quantitative Analysis

A total of 13 parents took part in the quantitative evaluation of the education program (response rate 65%). Of these 12 were female and 1 was male, and average age was 47.9 years. The questions were answered using a scale from 1 (highest grade) to 6 (lowest grade) (see Supplementary Tables S4 and S5 ).

Overall, parents rated the education program very highly (1.8 ± 1.0). They rated their knowledge after the program (1.5 ± 0.7) higher than before (2.8 ± 0.7). The individual modules of the program were generally rated from good to very good, while the content considered novel was mostly rated as average. Particularly the possibilities for exchange (1.8 ± 1.0) and the online format (1.4 ± 0.5) were perceived as good, as well as the opportunities for personal notes and questions. One improvement suggested was to divide the group according to the severity of their children's illness. Additionally, participating parents expressed a desire for more interaction, more time for questions, and more scheduling options at different times.

#### Qualitative Analysis

The responses of parents formed the following six main categories: Characteristics of the participants, education program resources, (online) format, outcome, overall evaluation, and recommendation (see Supplementary Fig. S2 , available in the online version only).

#### Characteristics of Parents

The majority of the interviewed parents stated having comprehensive prior knowledge due to independent research or previous clinic stays. Expectations and motivation varied. Although most parents expected to gain new information about the illness from the program, the “exchange with other affected parents” was mentioned as the second most common expectation. Other parents had no expectations, hoped to learn more about the management of the disease, expressed the desire to support research, or participated because the study offered this opportunity.

#### Education Program Resources

Both the selection of content and participation opportunities within the study were satisfying for participating parents. The assessment of program materials, the website, and the facilitators were positive as well. The participants were also largely satisfied with the organization and the course of the program: “I found it well chosen in terms of timing that as a parent, I can take time in the evening with some lead time (…).”

#### (Online) Format

The online format was predominantly well received by participating parents. In particular, they appreciated time savings and the elimination of travel: “(...) you don't have to undertake any trips, and if we had to go to Würzburg every time, it would have been difficult. We have three children, and one affected child—making that possible every time would have been difficult.” Regarding the possibility of exchange in the online format, there was a mixed response. While some encountered difficulties, as expressed by one person, “You don't get into a conversation as easily; everything is a bit reserved,” another person reported that the exchange worked smoothly, and that they had “no problem.” The interviewed parents were generally satisfied with the format and the opportunities of participation.

#### Outcome

Although many contents were already familiar to the parents, and thus, not entirely new aspects were presented to many, the program was considered a good opportunity for a refresher by many respondents: “(…) that refreshed it, so that you thought: Ah yes, we tried that a year ago and then didn't do it anymore, we could try that again.” Particularly, the module on legal aspects was popular among the interviewed parents. Parents also reported incorporating program content into their everyday lives: “But now, of course, we also do a lot of what we learned in the program (...).” Furthermore, the program helped in dealing with the disease and improved understanding toward the affected individuals, for example, regarding pacing: “And then it's also easier for us to say okay, you know how far you can go. You said it's over, and then it's over.” Parents also reported feeling able to better support independent disease management of their children. They also expressed a sense of belonging after the program, stating that it was “comforting to see [that] you're not the only one in this situation, so there are other families, other children, other parents who face the same challenges as you do.”

#### Overall Evaluation

Overall, the interviewed parents expressed great satisfaction with the parent education program and that “you are simply taken seriously because that is sometimes a bit difficult in everyday life with this medical condition.”

The training in small groups of maximum 12 parents was rated very positively by the participants, especially regarding the opportunity “to exchange with each other,” both during the program itself and afterward in the form of the created chat groups, “that there is simply still the possibility, if necessary, to reach others and at least get an opinion.” These not only conveyed helpful recommendations but also a sense of belonging.

Improvement suggestions included, among other things, a more in-depth discussion of the topics, extending the time of sessions, fostering participant interaction at the beginning of the program, and tailoring the program to the prior experience or disease severity of the affected CYP.

#### Recommendation

All parents stated that they would recommend the program to other parents.

## Discussion

The results of the study demonstrate the feasibility, high acceptance, and overall positive evaluation of the online education programs for both CYP with ME/CFS and their parents.

### Acceptance

The education programs were well received among the participants, with high approval rates regarding recommendation. Expectations of the participants for the program were met. To our knowledge, other online ME/CFS education programs for CYP have not yet been established, making a direct comparison challenging. However, studies of ModuS programs for other conditions show comparable results. 11 21 Families, in general, were very satisfied with ModuS. For example, in a study by Menrath et al evaluating education programs addressing the topic of medical transition, CYP reported an average satisfaction score of 27.8 on the ZUF-8 scale (8 = very dissatisfied, 32 = very satisfied) and their parents rated the program with a score of 26.5. 11 Evaluating a ModuS education program for children with asthma, affected children rated the program on the ZUF-8 scale as between 1 and 6, with a mean score of 1.4 (SD 0.6), and parents evaluated the program with a mean score of 27.6 (SD 6.0). 22

### Format

Evidence indicates that digital health interventions at any age can improve patients' self-efficacy and facilitate effective management of chronic conditions. 23 24 Here, the online format was highly regarded due to its flexibility both by CYP and their parents. Affected CYP described the option of not using a camera during periods of high stress as very positive, as well as the elimination of travel which saved energy. This is particularly important because, in ME/CFS, even slight overexertion can trigger PEM. 2 Furthermore, some of the severely affected CYP would not have been able to leave the house and travel to participate. Therefore, the online format improved accessibility. This is particularly important given the frequent isolation of affected individuals due to their physical limitations. 5 Parents, however, often emphasized the organizational advantages, notably the time saved and the avoidance of travel. Although some CYP and parents perceived the online format as less personal, the majority stated that effective interaction was nonetheless achieved. One CYP expressed disappointment that many had their cameras turned off. However, it can be assumed that CYP who, in the present study, could only participate with their cameras turned off would likely not have taken part in any in-person program, thereby significantly increasing the participation rate. For future programs, maintaining strong opportunities for interaction, while accommodating the diverse energy resources of participants, will be essential.

As an improvement suggestion, some participants expressed a desire to divide groups based on prior experience or disease severity. However, creating different educational programs for distinct levels would require significant effort. Additionally, a homogeneous group of severely affected patients might benefit less from participation due to reduced engagement and interaction levels resulting from their limited energy. To address diverse needs, it might be beneficial to develop and distribute different materials for review based on the severity of ME/CFS.

### Content and Benefit

The selection of content and the provided materials were well received by both the affected CYP and the parents. This aligns with findings from other studies indicating that patients' knowledge of ME/CFS often does not originate from healthcare professionals but is instead acquired through personal experience and a process of “trial and error.” 25 Due to the variety of offered materials for affected CYP it seemed that there was something for everyone. The reported prior knowledge of affected CYP and their parents can be attributed to the recruitment of participants after at least one specialized medical and psychological consultation. This was also shown in the evaluation of the parents' program, with high grades regarding overall evaluation of the individual modules and lower grades for novelty content. Nevertheless, the majority of the surveyed affected CYP and parents reported learning or refreshing aspects in the program and particularly transferring content into their daily life. The education programs, therefore, seemed to have met the needs of those groups. In addition to knowledge transfer, learning to cope with the illness as well as, for parents, improved understanding of their child's condition and how to support it, were prominent outcomes. These effects of the program represent important aspects for promoting self-management, a central component of caring for chronically ill children and adolescents. 22 A sense of belonging associated with a well-received group atmosphere and opportunities to exchange was reported by CYP and their parents, which might be an important aspect regarding their frequently perceived isolation and stigmatization. 26 This may also help address patients' feelings of insufficient support, which stem from skepticism about the legitimacy of ME/CFS among healthcare providers and the general public. 25 The exchange led to the formation of an online chat group, which often continued afterward. As improvement suggestions, CYP wished for additional topics such as nutrition in ME/CFS, which is already planned in a subsequent project. On the other hand, parents rather wished for deepening topics, more time for questions and for refresher programs which might be important during the course of the disease. Some individuals affected by ME/CFS, on the other hand, criticized frequent repetitions. However, it should be noted that participants usually have varying levels of knowledge to some extent, and that frequent repetitions are key aspects of patient education programs. In programs addressing ME/CFS frequent repetitions seem important to support the practice of distinct strategies such as pacing.

## Limitations

This study has several limitations: The selection of interviewees may have been influenced by the fact that only motivated participants may have agreed to participate. However, to prevent only less severely affected participants from being included in the survey, care was taken to ensure that three interviewed individuals were severely ill.

Another limitation was the absence of a control group, which was primarily due to the small number of patients available. Despite this limitation, the promising results from this pilot study provide a strong rationale for conducting future research with a larger sample size and the inclusion of a control group to more rigorously evaluate the intervention. Furthermore, the response rate of the parent questionnaires, despite multiple reminders, was not optimal. It should be noted that parents are often under considerable stress, not only due to their affected children but also due to the demands of their unaffected siblings, and therefore often have limited time. Nevertheless, with the additional insights gained from the qualitative analysis, we believe that a sufficient evaluation of the education program can still be provided.

## Summary

This study highlighted the feasibility of online patient education programs for CYP with ME/CFS and their parents. The online format was well-received, primarily due to its flexibility, accommodating different forms and durations of participation, and saving time for parents and their children. It was widely regarded as providing ample opportunities for interaction. Despite having good prior knowledge, most participants gained new insights or refreshed their understanding of the disease, benefiting particularly from the group exchanges during and after the program. All interviewed participants expressed a willingness to recommend the program to others.
