# Supplementary material for: Evaluation of an Online Patient Education Program for Children and Young People with ME/CFS and their Parents within the BAYNET FOR MECFS Study
Source: Neuropediatrics. 2026 Jan 6;57(2):149–55. doi: 10.1055/a-2773-9655 (PMC12956383; doi:10.1055/a-2773-9655)
Supplement: Supplementary file 1 — Supplementary Material [file 10-1055-a-2773-9655-s0920254170oa.pdf]

Supplementary Material

Supplementary Table S1. Overview of the ME/CFS education program for children and adolescents with ME/CFS based on the ModuS curriculum (Total of 12 sessions, each session lasted 45 minutes, and participants were given the option to leave early if their energy levels were low. For more detailed information see Keicher et al.[15]).

ME/CFS online education programme sessions	Modules description	Duration
1) Introduction	Introduction and motivating education about the illness, treatment, and prognosis	45 Minutes
2-3) Breathing and relaxation exercises and strategies	Skills and motivation for low-symptom intervals and skills for regulating and avoiding acute crises: emergency management	2 x 45 Minutes
4-5) PEM/Crash	Motivating education about the illness, treatment, and prognosis<br>and skills for regulating and avoiding acute crises: emergency management	2 x 45 Minutes
6-7) Pacing	Skills and motivation for low-symptom intervals and skills for regulating and avoiding acute crises: emergency management	2 x 45 Minutes
8-9) Additional therapies	Skills and motivation for low-symptom intervals and skills for regulating and avoiding acute crises: emergency management	2 x 45 Minutes
10-11) Disease management	Disease management within the family system	2 x 45 Minutes
12) Open discussion	Conclusion	45 Minutes

[a] blue = generic modules, gray = diagnosis-specific modules; [b] unit includes topics from both cross-disease and disease-specific modules;

Supplementary Table S2. Overview of modules of the online ME/CFS education program for parents of children and adolescents with ME/CFS based on the ModuS curriculum For more detailed information see Keicher et al.[20]).

ME/CFS online education programme sessions	Modules description	Duration
1) Disease pathogenesis and coping	Introduction and motivational education on illness, treatment, and prognosis, disease management within the family system	90 Minutes
2) Breathing and relaxation exercises and strategies & Pacing	Skills and motivation for low-symptom intervals and skills for regulating and avoiding acute crises: emergency management	90 Minutes
3) Therapy options & management of disease	Motivational education about the illness and skills for regulating and avoiding acute crises: emergency management and disease management within the family system	90 Minutes
4) Social legal aspects & school	Skills and motivation for low-symptom intervals and skills for regulating and avoiding acute crises: emergency management	90 Minutes

[a] blue = generic modules, gray = diagnosis-specific modules; [b] unit includes topics from both cross-diseases and disease-specific modules

Supplementary Table S3. Interview questions for affected children/adolescents with ME/CFS who participated in the education program.

Question block	Questions
1	• What were your expectations for the education program?<br>• Share your experience with the program. You can provide information about technical access, materials used, preparation materials, group size, atmosphere during the program, etc.<br>• Did you use the program materials, and did you find them helpful?<br>• Did you learn something new during the program?
2	• How understandable were the contents for you?<br>• Did you find the selection of topics suitable for you?<br>• What did you think about the online format?<br>• How did you like the videos on circulatory disorders, sleep disorders, and pain? (If you haven't watched the videos, you can skip this question)<br>• What was the most important thing you took away from the program?
3	• How has your daily life changed as a result of the program?<br>• Did you find the tips for improving sleep disorders helpful? (If you don't have sleep disorders, you don't need to answer this question)<br>• Did you find the tips for improving pain helpful? (If you don't have pain, you don't need to answer this question)<br>• Did you find the tips for improving circulatory disorders helpful? (If you don't have circulatory disorders, you don't need to answer this question)<br>• How did the exchange with other affected children and teenagers go?<br>• How well were you able to participate in the program?
4	

	• Did participating in the program overall prove worthwhile for you? (If yes, what factors contribute to this?)<br>• Would you recommend the program to a friend?<br>• What did you particularly like about it?<br>• What could be improved? (Feel free to mention any points that are important to you and haven't been addressed yet)

Supplementary Table S4. Semi-structured interview guide for parents' interviews.

1. Why did you originally sign up for the education program?
2. What specific expectations did you have for the education program?
3. What are your overall thoughts on the online format? What advantages and disadvantages do you see?
4. How would you assess your personal benefit?
5. How did you perceive the selection of content, the topics covered in the program? Did you find them relevant or not so relevant? Did you think they were well chosen or not so well chosen?
6. Were there any contents in the program that were new to you?
7. What overall impact did this program possibly have on your daily life?
8. How has your perception of the illness changed through the education program, specifically regarding this illness, ME/CFS? Or what influence did the education program have?
9. And did that perhaps also have an influence on dealing with you affected child/adolescent?
10. How did you experience the opportunity for exchanging contacts with other parents during the education program?

11. What do you think about the group size?
12. Regarding the contact opportunities, would you generally wish for something like that to continue, where you stay in touch with the other parents?
13. What was the most important thing you took away from the program? What was the most important aspect for you during the education program?
14. Would you say overall that you are satisfied with the education program?
15. Would you yourself also participate in further education programs for parents of children with ME/CFS?
16. What did you overall find good about the education program?
17. From your perspective, what aspects of the education program do you think could be improved?"
18. Regarding the materials you received during the program, do you think there's anything we could improve there?
19. Would you recommend the program to other parents?

Supplementary Table S5. Assessment of individual modules of the online parent education program (1=very good, 6=very bad, SD=Standard deviation).

**Questions**	**Average school grad±SD**
How do you evaluate your knowledge prior to the seminar?	2.8±0.7
How do you evaluate the selection of contents?	1.8±0.7
How do you evaluate the materials?	1.7±0.6
How do you evaluate the scope of the program?	2.2±0.9
How do you assess the benefit of the training for your daily life?	2.2±0.9
How do you evaluate the clarity of the program?	1.3±0.5
How do you evaluate your knowledge gain?	2.0±1.2
How do you rate the opportunity for your own comments, questions, and discussions?	1.3±0.6
How do you evaluate the opportunity to exchange with other parents?	1.8±1.0

How do you evaluate the design of the presentations?	1.4±0.5
How do you evaluate the group atmosphere?	1.5±0.6
How do you evaluate the group size?	1.5±0.5
How do you rate the program overall?	1.8±0.9
How do you evaluate the online format?	1.4±0.5
How likely are you to integrate the training content into your daily life?	1.8±1.0
How do you rate the fulfillment of your expectations of the program?	2.2±0.9
How well do you feel informed about ME/CFS after the training?	1.5±0.7
What did you particularly like? What did you not like at all? Where is there room for improvement?	Free responses
Would you recommend the training to other affected parents?	Free responses
Would you participate in further training on ME/CFS?	1.2 ±0.4
How do you evaluate the program module on the origin and processing of the disease?	1.7±0.6
How new were the contents of the program module on the origin and processing of the disease for you?	3.8±1.2
How do you assess the usefulness of the contents of the program module on the origin and processing of the disease for your everyday life?	2.5±0.9
How do you evaluate the program module on breathing and relaxation techniques as well as pacing?	1.9±1.0
How new were the contents of the program module on breathing and relaxation techniques as well as pacing for you?	3.7±0.8
How do you assess the usefulness of the program module on breathing and relaxation techniques as well as pacing for your everyday life?	2.6±1.0
How do you evaluate the program module on therapy options and disease management?	2.2±1.0
How new were the contents of the program module on therapy options and disease management for you?	3.3±0.8

How do you assess the usefulness of the contents of the program module on therapy options and disease management for your everyday life?	2.6±1.0
How do you evaluate the program module on school and social legal aspects?	1.9±0.8
How new were the contents of the program module on school and social legal aspects for you?	3.1±0.7
How do you assess the usefulness of the contents of the program module on school and social legal aspects for your everyday life?	1.8±0.8

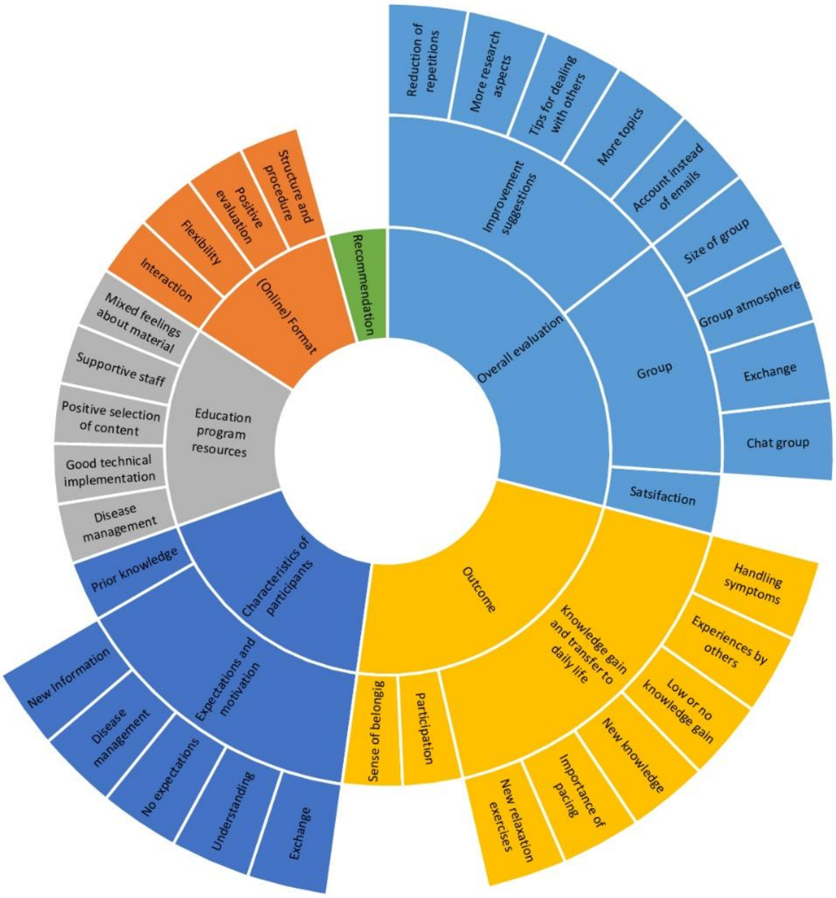

Figure 1: Overview of categories for the online education program for children and adolescents with ME/CFS (Colors mark related categories, main categories in the inner circle with outward arranged respective subcategories and sub-subcategories)

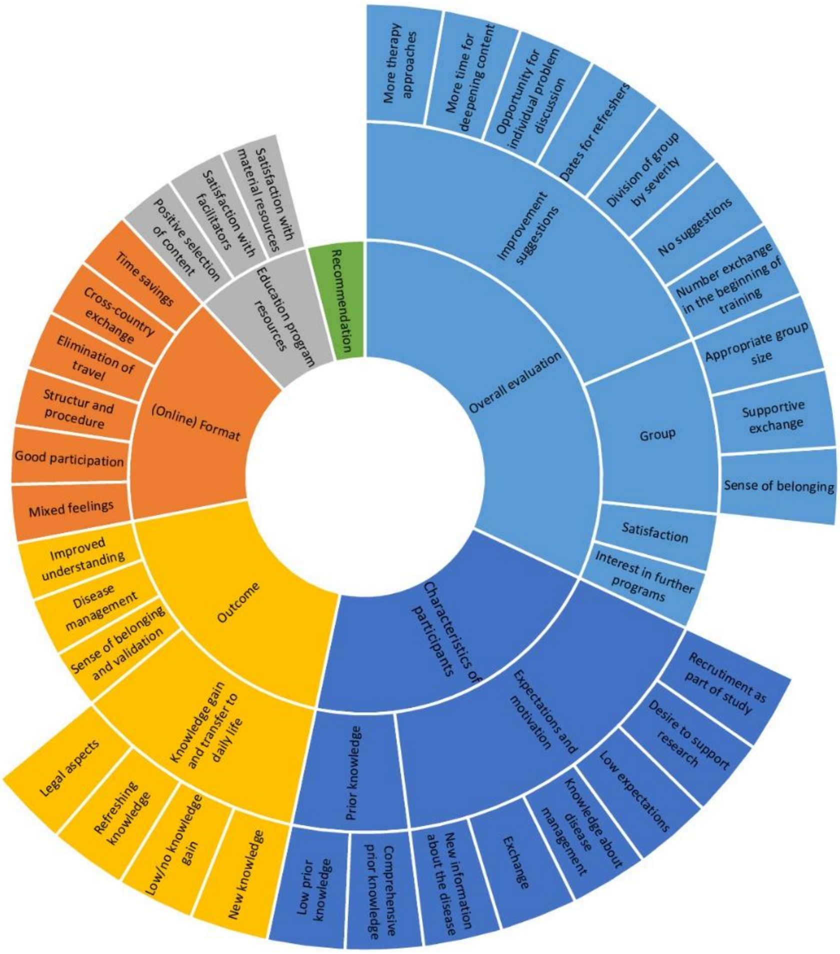

Figure 2:Overview of categories for the online education program for parents of children and adolescents with ME/CFS (Colors mark related categories, main categories in the inner circle with outward arranged respective subcategories and sub-subcategories)
